# Collectanea

**Published:** 1826-07

**Authors:** 


### COLLECTANEA.

Floriferus, ut apes, in saltibus omnia libant,
Omnia nos, itidem, depascimur aurea dicta.

### PHYSIOLOGY.

### *Observations on the Brain and Nerves in Monsters.*

M. Tiedemann, whose name is so well known as a distinguished anatomist and physiologist, has devoted much attention to the correspondence between certain imperfections in the brain and nervous system, and certain coexistent peculiarities in other parts, with a view of ascertaining whether the former have any influence in the production of the latter. He puts the following questions, and then offers some observations in reply.

1. When an organ is wanting, is there likewise a corresponding deficiency of the nerves?

2. When there is an excess in the number of the organs, is there a corresponding excess in the number of nerves, of the relative parts of the brain, and of the spinal cord?

3. What are the alterations of the nervous system which correspond to the imperfections in the development of the organs?

4. Is there a particular organisation of the nervous system, especially of the brain and spinal cord, which always corresponds to an anomalous organisation of the body in general, or any particular parts: and if so, in what does this consist?

The author has recorded eight observations, which relate to three different errors of organisation, and are regarded as throwing some light upon these questions.

1. *Congenital cleft palate, with defective organisation of the brain, and absence of the olfactory nerves.*—The particulars are these: An infant, who died immediately after birth, had hare-lip and division of the palate. On examining the brain, the hemispheres were found united at the fore part, the convolutions passing from one side to the other without any interruption. The olfactory nerves were completely wanting, and, in place of the ethmoid bone, there was a cartilaginous mass without perforations. The optic thalami were united at their upper part, and formed a sort of bridge over the third ventricle; the fornix was imperfectly developed.

A second case was identically similar, and a third nearly so; the brain having no division into hemispheres, and the olfactory nerves being absent. Analogous instances are to be found in the writings of Soemmerring and Rudolphi.

2. *Absence of the eyes and their nerves.*—M. Tiedemann saw a dog without eyes, the orbits being filled with cellular membrane. On examining the brain, two very soft threads were found in the place of the optic nerves: they arose from the optic thalami, and from the tubercula quadrigemina: they made a turn round the peducles of the brain, and terminated, without uniting, in front of the pituitary gland. The other nerves of vision were entirely wanting. Similar cases are recorded by Malacarne, Osiander, Lobstein, and others.

3. *Union of the eyes*; *anomalous formation of the brain.*—A fœtus at the full time, had no nose nor any organ of smell; there was only one eye, and this had four eyelids; the globe appeared double behind, and had an oblong shape in front. The hemispheres of the brain formed a single mass, without any trace of convolutions; the olfactory nerves were entirely wanting, as well as the ethmoid bone; the optic nerves entered the orbit without uniting.

Several other cases are related, from which it appears that the union of the two eyes is always accompanied with the absence of the organ of smell, the fossæ nasales, the ethmoid, the vomer, and the ossa lachrymalia. In many there is no mouth. In every case the tongue is wholly or partially wanting. The hemispheres of the brain are united, so as to form a single mass, less in size than the usual dimensions of the cerebrum; and the surface generally presents no appearance of convolutions. The corpus callosum is not formed. The olfactory nerves do not exist in those monsters, who have not the organ on which they are usually distributed; and their absence is accompanied by a diminution in the size of the corpora striata, and the absence or imperfect development of the fornix and cornua ammonis. The optic nerves generally unite before they enter the orbit, although they sometimes enter separately, and in no case is there any communication between them.

From these and other instances Tiedemann concludes, that the configuration of the brain, and the arrangement of the nerves,—is intimately connected with the development of the respective organs, and that the nerves do not exist when the organs are not developed; and likewise that the development of the bones is in direct relation to that of the organs they are intended to inclose. (*Zeitschrift für Physiologie*)

---

### PATHOLOGY.

### *Extraordinary Case of Hydrocephalus.*

Excepting the case spoken of by Bartholin, wherein the head was four feet in circumference, a case related by Dr. Goebel probably affords an example of one of the largest heads mentioned in the records of medicine. The child was born hydrocephalic, and is now six years old. At one year old, the head was nearly as large as it is at present. The size is so great, that the left ear is situated horizontally: the superior fontanel is five inches across. The hair has never grown at all. The whole body is extemely emaciated, and forms a singular contrast with the size of the head. The child weighs thirty-four pounds altogether, the head weighing twenty-eight alone. The largest diameter of the head is fourteen inches two-thirds, and the greatest circumference thirty-one inches and a half. The rotundity of the cranium is not uniform, but presents various protuberances. The child eats well, and begins to speak imperfectly; his intellectual faculties appear to be tolerably developed. Both the urine and stools pass involuntarily. His sleep is profound, and but of short duration. (*Annali Universali.*)

### *Disease from Spurred Rye.*

Mr. Theodore F. King, house-surgeon of the New York Penitentiary, describes thirty-two cases of morbid affection from this cause, occurring in that prison, in the fall of last year. Small livid spots made their appearance on one or both of the lower extremities, generally about the foot, without any local pain or weakness, or any perceptible general indisposition. After a few days, they would become more numerous, and extend up to the knee. Pain now occurred,—in one case very severe; and in some instances the patient lost the use of his lower extremities. The pulse was feeble, and seldom exceeded 100 in a minute. The face had a peculiar livid appearance. No unusual heat of the skin occurred. The tongue was slightly coated, and "very flabby." In several cases, there were considerable sores about the mouth, and in two or three hemorrhage from the gums; bowels generally regular; urine natural; death always preceded by "severe colicky pains;" usual duration of the disease about four weeks. Ten cases proved fatal. No mention is made of uterine pains occurring among sixteen females.

The only dissection made exhibited the intestines studded with dark livid spots, in the greatest abundance upon the large intestines, and the stomach inflamed, with a discoloration upon the under and larger portion. The uterus was natural.

Has any one ascertained whether the American ergot be or be not a different species from that of Europe? We have here, among thirty-two patients, not a single case of gangrene recorded. (*New York Med. and Phys. Journal.*)

---

### PRACTICAL MEDICINE.

### *Prussic Acid.*

The hydrocyanic (prussic) acid is a valuable medicine, but requires, according to my observation, more care in the administration of full doses, than any other medicine. It may be given in small doses, usually, without any inconvenience. When its qualities were first proclaimed, it was too highly extolled as a remedy in phthisis pulmonalis; and, from consequent disappointment, I conceive that its just merits are not sufficiently appreciated. In some cases of hectic fever, attended with urgent cough, I have procured the happiest effects from the use of this medicine. I have not, with the adult patient, in any instance, prescribed more than twenty-four minims as the total quantity in twenty-four hours; and usually have confined myself to the extent of fifteen, always commencing with small doses. (Dr. SCUDAMORE's *Work on the Stethoscope, &c.*)

### *Leeches.*

The application of leeches, with a view to derive blood from the vessels which communicate with those of the lower bowels, is a practice quite common in France, and seems a favourite measure, whatever viscus of the body may be affected. I have no doubt of the utility of this mode of obtaining blood, when the lower part of the intestinal canal is in a state of congestion; and I may add, in many cases of irritation. A gentleman had been troubled with diarrhœa, which was often painful, for two or three months. It had resisted the usual treatment by medicine. By one application of leeches near the rectum he was cured. (*Ibid.*)

---

### STATISTICAL MEDICINE.

### *On the Duration of Human Life in France.*

At a late meeting of the Royal Institute of France, M. FOURIER read a very interesting Memoir, by M. BENVISTAN DE CHATEAUNEF, on the Changes that the Laws of Mortality have undergone, from 1775 up to 1825. The Memoir contains a great many curious and interesting details, of which the following are a few:

| | | |
|---|---|---|
| In 1775, of every 100 children, | 50 | died before two years old. |
| In 1825, —— | 38.3 | |

This difference may in a great measure be attributed to the introduction of vaccination.

| | | |
|---|---|---|
| Formerly, of every 100 children, | 55.5 | died before ten years old. |
| In the present day, out of the same number . . . . . . . . | 47.7 | |

Formerly, of every 100 male children, only 21.5 arrived at fifty years of age;
In the present day, out of the same number, 32.5 come to fifty.

In examining the other epochs of life, and comparing them, the comparison is always in favour of the present time.

| | |
|---|---|
| Formerly the Mortality was annually | 1 in 30, |
| Now it is only . . . . . . . | 1 in 39. |
| Formerly the Births were annually | 1 in 25, |
| Now . . . . . . . . . . | 1 in 31. |
| Formerly Marriages were annually | 1 in 111, |
| Now . . . . . . . . . . . | 1 in 135. |

The fecundity appears to have been the same formerly as at the present time; the births, as well as deaths, have diminished; and the term of human life is longer. One may discover a cause of the diminution of births in the fewer marriages that now take place; but the number of foundlings is more than tripled since 1780. Population, however, must increase, because the term of life is longer; and it is the duration of life that must increase it, rather than the birth of a few children more, of whom death cuts off 48 in every 100 before the age of two years.

The difference in the population of France is also given, being the result of a mean of ten years for the first epoch, and of eight for the second.

| | | | | |
|---|---|---|---|---|
| In 1780, | the Population was | 24,800,000; | in 1826, | 30,400,000, |
| — | Deaths — | 818,490 | — | 761,230, |
| — | Births — | 963,200 | — | 957,970, |
| — | Marriages — | 213,770 | — | 222,570, |
| — | Natural Children, | 20,480 | — | 65,760. |

The Mortality at different ages was as following:

| | | | | | | |
|---|---|---|---|---|---|---|
| In 1780, | from birth to | 10 years of age, | in 100, | 55.5; | in | 1825, 43.7, |
| — | — | 50 | — | 78.5 | — | 67.5, |
| — | — | 60 | — | 85 | — | 76. |

It is thus shown that the lot of mankind, with regard to the mean duration of life, has prodigiously increased in France.

(*Revue Medicale.*)

---

### SURGERY.

### *Case of Spina Bifida in the Neck, cured by Puncture.*

M. Labonne has recorded a case of this kind. A child was born with a tumor on the cervical vertebræ, as large as an orange. It was moveable, with red spots on the surface, and was of equal dimensions at every point, even the base. Various stimulating applications were resorted to; notwithstanding which, the tumor increased in size. At the end of a year, the infant was lively, and appeared well; but M. Labonne, urged by the entreaties of the mother to do something for her child, made five little punctures at the sides of the tumor, three days after the administration of a gentle purgative. A lemon-coloured fluid escaped, and continued to ooze for eight days: the tumor then diminished. Emollient cataplasms were laid over the part, and slight compression applied to the head. An eruption supervened; little papulæ, like flea-bites, appeared on the spine; they suppurated, and the tumor entirely subsided. The writer concludes with some observations on the superiority of the puncture, in such cases, over cautery, caustic, compression, and the other methods which have been proposed. (*Ibid.*)

### *On Amputation at the Knee-Joint.* By Dr. Nathan Smith, Professor of Surgery, &c. Yale College.

For several years I had contemplated amputating at the knee-joint, whenever a case should occur in which it would be imprudent to operate below the knee, on account of disease in the bones or soft parts, and in which the superior part of the articulation was sound. I had often performed the operation on the dead subject, and found that it might be so accomplished as to leave a very good stump.

A case warranting the operation occurred to me in April, 1824. Miss R. D—, of Brunswick, Maine, having a disease in both the bones and soft parts of the leg, of long standing, and so extensive that it was thought improper to operate below the knee; and, as the inferior part of the femur was sound, I amputated at the joint.

The operation is performed as follows:—Mark two points, one on the out and the other on the inside of the limb; the latter half an inch below the head of the tibia, and the other opposite to it.

Then draw a semicircular line from one point to the other, over the anterior part of the leg, and in such a direction that its lower part shall touch the lower part of the tubercle on the tibia into which the ligament of the patella is inserted, and then mark another circle on the posterior part of the leg, exactly corresponding to the former. The above lines limit the two flaps, the former of which will be formed of the patella and its ligament, together with the investing integuments; and the latter of the head of the gastrocenemeus, the tendons of the flexor muscles, and the popliteal blood-vessels and nerves. The operator should first raise the anterior flap with the patella, which will expose the anterior part of the joint, and render the division of the lateral ligaments easy. Two or three strokes of the knife will then complete the section of the lower flaps, with the crucial ligaments.

In the case mentioned above, the patient recovered without the occurrence of any thing unpleasant. It was accomplished with less pain to the patient and trouble to the operator, than when the separation of a bone is required. The stump will obviously be more useful, as the lower part of it is formed by the patella, which becomes anchylosed to the femur.

This operation has been deprecated by many, on account of the complicated structure of the joint, and because of the violent and often destructive inflammations which result from wounds of the knee. By the operation, however, the most complicated part of the apparatus is removed, and the motions of the joint, which is the unfavourable circumstance in wounds of that part, do not irritate the wound. The synovial membrane immediately assumes the adhesive inflammation, and union is speedily effected. (*American Medical Review*, No. 2.)

*Operation for Phymosis.*

Having observed, in the last Number of the London Medical and Physical Journal, a notice of an operation, recommended, and said to be new, by M. J. Cloquet, of Paris,—namely, the operation for phymosis, by dividing the prepuce in a direction parallel to the frenum,—I am induced to state that, in 1817, I performed that operation upon a young officer in the public service; and that I

have since repeated it, in 1824, upon a private patient. The first case originated in natural defect, the second in diseased action.

It has often astonished me that the expediency of this improvement had not suggested itself to surgeons of experience, who must have frequently witnessed the unseemly purse-like thickening of the prepuce, after the ordinary mode of operating in a line with the centre of the dorsum penis. Here, as is natural, the divided prepuce, distended by blood effused into its cellular texture from the bleeding vessels, contracts and gravitates, forming a tumor which never completely subsides; and sometimes demands amputation, from the inconvenience with which it is attended.

The instrument employed in the above cases was a sharp-pointed bistoury, which, being passed through the prepuce at its attachment with the glans, was afterwards carried with one stroke through the included parts; a mode which I consider very preferable to that of employing scissors, which must contuse and injure the parts in cutting them. (*Note from* Mr. BOYLE.)

*4, Cleveland-square, St. James's;*

*1st of June, 1826.*

### *A Case of Destruction of the Epiglottis, in which Life was prolonged for several Months.*

—— Miller, in Cable-lane, in the year 1802, was afflicted with disease of the larynx, attended with ulceration, which had nearly destroyed the epiglottis, as appeared on examination after death, by Drs. HEWSON and ROUSSEAU: the impossibility of swallowing liquids exposed him to suffocation. His solid food was made into a ball, in order to prevent its descent into the glottis; and thus he protracted his miserable existence. His drink was supplied by a catheter introduced into the stomach, through which was injected water, broth, alcohol, and other liquids: as this process was troublesome, and required the attendance of the physician, he was necessarily left to the care of his wife. By the advice of Dr. Rousseau, the intestine of a chicken was directed to be swallowed by the patient, one end of which was fastened to one of his teeth by a ligature, and the other end terminated in the stomach; through which his wife injected broth and other fluid articles of nutriment, and supported him for several months: the intestine was changed as often as necessary. (*American Med. Recorder.*)

### *Extirpation of an Ovarium.* By ALLAN G. SMITH, of Danville, Kentucky.

In this case the ovary, which was scirrhous, was extracted by an incision from the umbilicus to the pubis: the tumor was removed, previously surrounding it with a ligature of silk. The wound was closed with the interrupted suture, and the ligatures came away by the twenty-fifth day. The woman gradually recovered. (*Ibid.*)

### MISCELLANEOUS.

### *Snails as an Article of Food.*

M. de Martens states, that the annual export of snails (*Helix pomatia*) from Ulm, by the Danube, for the purpose of being used as food, in the season of Lent, by the convents of Austria, amounted formerly to ten millions of these animals. They were fattened in the gardens in the neighbourhood. This species of snail is not the only one which has been used as food; for, before the revolution in France, they exported large quantities of the *Helix asperso* from the coasts of Aunis and Saintonge, in barrels, for the Antilles. This species of commerce is now much diminished, though they are still sometimes sent to the Antilles and Senegal.

The consumption of snails is still very considerable in the departments of Charente Inferieure and Gironde. The consumption in the Isle de Rhé alone is estimated in value at 25,000 franks; and at Marseilles the commerce in these animals is considerable. The species eaten are *Helix rhodostoma*, *H. aspersa*, and *H. vermiculata*. In Spain, in Italy, in Turkey, and the Levant, the use of snails as food is common. It is only in Britain that the Roman conquerors have failed to leave a taste for a luxury which was so much used by the higher classes in ancient Rome; though it would be very desirable, for the sake of the produce of our gardens, that some of the leaders of fashion in eating would, by introducing them at table, take the most effectual method of keeping our native species within due bounds. (*Edin. Jour. of Science.*)

